# A Path-Planning Scheme for Autonomous Vehicle Navigation Integrating BJA* and Improved DWA Algorithms

**DOI:** 10.3390/s25227017

**Published:** 2025-11-17

**Authors:** Kai Xin, Guoxu Zhou, Huacai Lu

**Affiliations:** 1 Anhui Key Laboratory of Detection Technology and Energy Saving Devices, Anhui Polytechnic University, Wuhu 241000, China; 15851885578@163.com; 2 School of Electrical Engineering, Anhui Polytechnic University, Wuhu 241000, China; z19339169529@stu.ahpu.edu.cn

**Keywords:** A* algorithm, BJA* algorithm, DWA algorithm, navigation path planning

## Abstract

Aiming to address the defects of the traditional A* algorithm such as mismatch with dynamic environments, insufficient path smoothness and poor real-time obstacle avoidance and the limitations of the traditional DWA (Dynamic Window Approach) algorithm, which easily falls into local optima and relies on parameter tuning, this paper proposes an autonomous driving vehicle navigation path-planning scheme fusing BJA* (Bidirectional Jump point A*) and improved DWA. The fused algorithm enhances A*’s global efficiency via a 24-neighborhood search and a bidirectional jump-point strategy and boosts DWA’s local robustness by optimizing the evaluation function and integrating global path information. MATLAB (2022b)-based simulation experiments were conducted. In global path planning, BJA* was compared with improved A* methods, post-processed for path planning, and evaluated through ablation experiments that highlighted the contribution of the improvements; in local obstacle avoidance, vehicle posture, linear and angular velocities under different dynamic scenarios were compared. Experiments show that BJA* exhibits obvious improvements in path length, traversal time and turn number and that integrated local obstacle avoidance makes equipment speed control run more stably.

## 1. Introduction

The planning module of an autonomous vehicle is key to its operation, and successful path planning generates a path that is efficient and safe. An optimal path from the starting point to the target point is planned with the goal of avoiding various obstacles [1,2]. Path planning can be divided into two parts: global planning and local obstacle avoidance. Global planning refers to the macro-level optimal path that is planned when the vehicle considers the static elements of the external environment as static before starting to drive, generates the starting point and end point, and generates a path that runs between those points [3]. Commonly used global path-planning algorithms include Dijkstra [4], A* [5,6], RRT (Rapidly-exploring Random Tree) algorithm [7], GA (Genetic Algorithm) [8], ACO (Ant Colony Optimization) [9], and PSO (Particle Swarm Optimization) [10]. Local obstacle avoidance relies on the vehicle’s global path data, which change according to changes in the environment to ensure fast and safe navigation. Commonly used local obstacle-avoidance algorithms include APF (Artificial Potential Field) [11] and DWA (Dynamic Window Approach) [12] and other local obstacle-avoidance algorithms. It is difficult for a single path-planning algorithm to cope with complex and dynamic actual road conditions, and it is necessary to integrate information from multiple sources and multiple algorithms to make up for the limitations of traditional algorithms and generate fusion algorithms for more reliable and intelligent path planning. At present, popular fusion algorithms include CDBA-RRT* [13], MA-HCAGA [14], and AOC-A* [15].

The A* algorithm is one of the most commonly used algorithms for global path planning, and a large number of researchers have improved it [16]. However, paths planned by the A* algorithm have the shortcomings such as proximity to obstacles, excessive node exploration, overly tortuous trajectories, and a lack of dynamic obstacle avoidance. Kong [17] improved the search efficiency of the path by improving the evaluation function of the A* algorithm and introducing a two-way search mechanism but did not consider dynamic changes in the scene. Zhang [18] proposed a directional weighted A* improvement algorithm to effectively plan a relatively smooth shortest path from the starting point to the end point that avoids obstacles, but these planned paths may diagonally cross the apexes of obstacles. Xu [19] used the two-way rectangular expansion and slide-rail corner-adjustment method to improve the A* algorithm; this approach shortened the search time and improved the path smoothness but did not consider the complex environment and did not include dynamic obstacle avoidance. Chen [20], aiming to address the problem that the paths often cross obstacles diagonally, generated a solution based on the A* algorithm, where local path constraints are added to the forward- and reverse-searched paths and cubic B-splines are introduced to optimize the inflection points in the path; however, the A* algorithm cannot adjust the path in real time when faced with dynamic obstacles.

DWA is the most widely used local obstacle-avoidance algorithm. It generates short-term trajectories and evaluates them within the feasible velocity window to achieve real-time obstacle avoidance, but it has the disadvantages of insufficient adaptation to dynamic obstacles, strong dependence on parameter tuning, and a strong tendency to fall into local optima. The improved DWA algorithm proposed by Sun [21] can plan a smooth path and has obstacle-avoidance functions, but it is ill-suited to handling fast-moving and sudden changes of direction, and it struggles to avoid obstacles in time. The improved DWA algorithm proposed by Chang [22] optimizes the speed space with the aim of improving obstacle avoidance, but parameters such as safety distance, speed weight, and evaluation function coefficient need to be manually debugged, the universality is poor across different scenarios, and there is no unified optimal solution. Missura [23] proposed a DWA-improvement method based on conflict prediction, which reduces the number of conflicts in a dynamic environment, but it lacks global consideration and is prone to falling into the local optimal solution and deviating from the global optimal path.

The A* algorithm does not include dynamic obstacle avoidance, and the DWA algorithm lacks global vision. Fusion methods incorporating global vision and local obstacle avoidance are often used in path planning for autonomous driving, but there are problems such as high algorithmic computational complexity and tension between path smoothness and motion constraints. Liu [24] proposed a fusion algorithm combining A* and DWA that improves the obstacle-avoidance ability, but the global search of A* traverses a large number of nodes, and the dynamic window sampling of DWA frequently simulates trajectories, significantly increasing the consumption of computing resources after fusion and thus affecting the real-time performance of autonomous driving. The A* and DWA fusion algorithm proposed by Wu [25] plans the path in real time, which reduces the number of traversal nodes and smooths the trajectory curve to a certain extent, but the path generated by A* may contain oblique vertices or unnecessary turns and thus may need to be further optimized. The trajectory fitting of DWA may sacrifice some path optimality, balancing safety and efficiency.

In this study, a combination of BJA* and DWA algorithms is proposed to address the above shortcomings, and it shows significant advantages in a number of performance indicators. The BJA* algorithm improves the adaptive search strategy of A* and the search method of bidirectional hopping points, reduces redundant nodes, balances global and local planning with adaptive weights, and also enhances the control of path smoothness and orientation, so as to efficiently generate global trajectories. The improvement of the evaluation function of the DWA algorithm introduces correction constraints such as obstacle density and path fit to improve the adaptability of DWA to complex environments, effectively balance the conflict between obstacle-avoidance safety and travel efficiency, and avoid the problem of motion stagnation caused by excessive conservatism in traditional methods. The algorithm-fusion mechanism enables the overall system to have both global planning and local adjustment capabilities, which significantly enhances the overall robustness of task execution and quality of trajectory generation of autonomous vehicles, providing reliable support for the application of this technology in real environments.

## 2. Materials and Methods

### 2.1. BJA* Algorithm

#### 2.1.1. 24-Neighborhood Search

**Figure 1:**
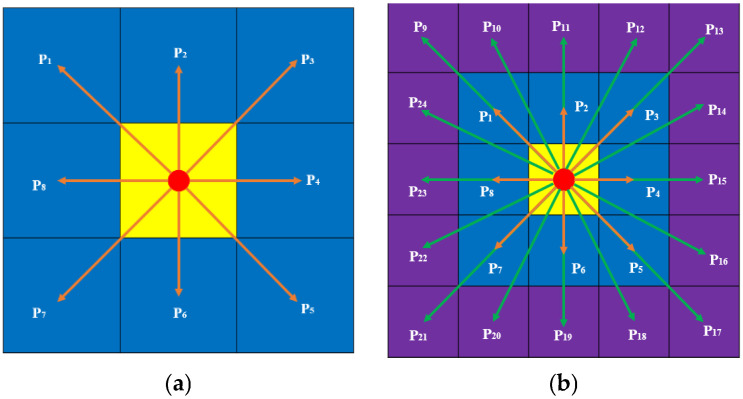
Optimization of node search methods. (**a**) 8-domain search. (**b**) 24-domain search.

In the process of path planning with the traditional A* algorithm, it is necessary to traverse eight neighborhoods around the current node, namely P_1_ to P_8_, with a direction angle range of [0°, 360°] and an integer multiple of 45°. The central node is used to calculate the path cost of all passable nodes in the eight neighborhoods, and the neighborhood node with the smallest path cost is taken as the central node for the next expansion search [26]. Due to the limitation of the forward-direction angle, the path planned by the traditional A* algorithm using eight neighborhoods may have a large number of turns and a relatively high path cost. In response to the above issues, the design has further expanded the search range to sixteen more neighborhoods beyond the original eight-domain search, adding P_9_ to P_24_. The range of the moving direction angle remains [0°, 360°], breaking through the limitation that the steering angle must be an integer multiple of 45°, a change that directly enables autonomous vehicles to move more flexibly. The eight-neighborhood and twent-four-neighborhood search diagrams are respectively shown in Figure 1a,b.

**Figure 2:**
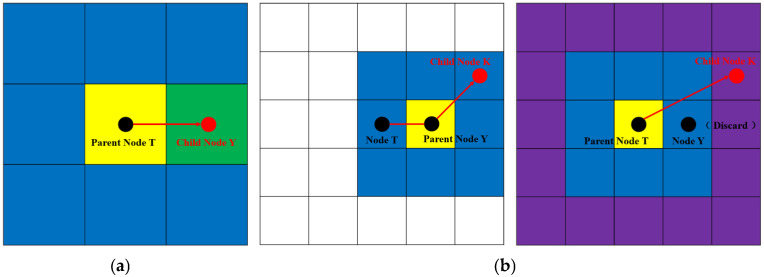
Principle underlying optimization of the node-search strategy. (**a**) Before optimization. (**b**) After optimization.

Let the cost of moving one node laterally or vertically be 10. The estimated cost distance is calculated using the Manhattan distance formula. Before optimizing the node-search strategy, search in eight surrounding directions with T as the parent node. Assume that node Y is the optimal node and is denoted as the child node Y. Then, conduct the same eight-neighborhood search conducted at node T with child node Y as the parent node. Assuming that the child node of node T is K, the number of paths turns from node T to node K is 1 and the path cost is 24. This process is shown in Figure 2a. After the node-search strategy has been optimized, when it has been determined that the parent node T has no obstacle restrictions in searching the surrounding eight neighborhoods and that all the surrounding nodes are passable, the parent node T no longer needs to calculate the optimal node but instead continues to expand the search domain by another 16 areas. Assuming the child node is K, the number of turns from node T to node K is 0 and the path cost is 22. This process is shown in Figure 2b. A comparison of the node-search strategies before and after optimization shows that the improved optimization strategy reduces the number of turns and the path cost.

#### 2.1.2. Jump-Point Selection

In a raster map, the Euclidean distance between the starting point and the target point is the theoretical shortest path. When there are obstacles between the two points, vehicles need to bypass the obstacles to move forward, thus making the search process more complicated. The traditional A* algorithm generates a large number of redundant computations in large-scale or open grids. Many nodes have no practical significance for path optimization. In fact, only the nodes along the straight-line direction are meaningful points for vehicle path planning. To solve this problem, “skipping meaningless intermediate nodes and focusing only on the skip points” was proposed, where the “skip points” refer to the key nodes. The selection of jump points requires preprocessing the obstacles that the straight-line SG passes through, extracting their discrete node coordinates, dynamically marking the nearest passable nodes along the straight-line direction, calculating the distances between nodes to determine the distribution of static obstacles in the straight-line direction, eliminating the corresponding dense nodes, and laying the foundation for the subsequent algorithm work.

**Figure 3:**
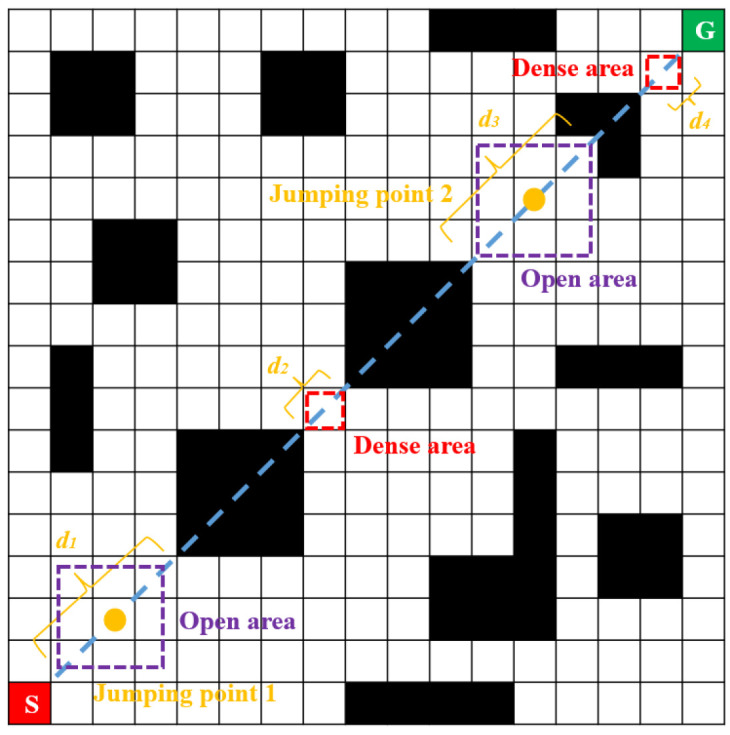
The process of selecting jump points.

First, construct the equation of the straight-line SG, traverse all the coordinate points of the squares that the straight-line SG passes through, detect the obstacles on the path, and record their coordinates. Search for the nearest passable node in front of and behind the obstacle in a straight-line direction and calculate the three Euclidean distances d in sequence. These are the Euclidean distances *d*1 (the distance between the two passable nodes of the obstacle), *d*2 (the distance from the starting point to the first passable node in front), and *d*3 (the distance from the last passable node behind to the end). Since the maximum distance between the centers of two grids is 14 and the minimum distance between the centers of three grids is 20, the maximum distance between two passable nodes is two grids and the minimum distance between two passable nodes is three grids. The next step is to set the distance threshold. The selection of the distance threshold is based on the number of grids between two passable nodes. If the distribution of obstacles on both sides of the passable node is relatively dense, no jump points are sampled. If the obstacles on both sides of the passable node are distributed far away from each other, this area is designated an open area and a jump point is obtained at the midpoint of that open area. The process of selecting jump points is shown in Figure 3. There, S represents the starting point, G represents the target point, black units indicate obstacles where vehicles cannot pass, white units represent areas where vehicles can pass, and orange dots indicate the confirmed jump points.

#### 2.1.3. Determination of Meeting Nodes

The traditional A* search strategy is a one-way search from the starting point to the target point. One-way search will expand many invalid nodes, thus resulting in low search efficiency. To address this problem, a bidirectional search strategy was designed. The idea is to change the traditional one-way search mode to start the search from both the starting point and the ending point simultaneously. When the same node is explored in both directions, the path can be concatenated and this node is designated a meeting node. Compared with one-way search, especially when self-driving cars are traveling on long paths, it can reduce the number of nodes explored with ineffective expansion.

**Figure 4:**
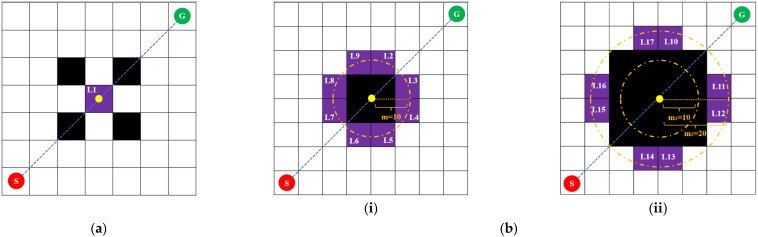
The process of identifying the meeting node. (**a**) The center is located in the passable area. (**b**) The center falls on an obstacle. (**i**) The step size is appropriate. (**ii**) The step size is insufficient.

The traditional bidirectional search strategy suffers from issues such as cumbersome encounter judgment and optimality-verification logic, as well as inaccurate *H*(*n*) estimation, which may make it difficult for the two searches to meet and increase the cost instead. It is rather difficult to determine the encounter point, and the judgment logic needs to take into account both “encounter identification” and “optimality verification” to avoid terminating too early, which leads to suboptimal solutions, and terminating too late, which wastes computing resources. An improved bidirectional intersection search strategy was proposed to address this problem, effectively solving the problem of determining the meeting node. The principle by which the meeting point is identified is shown in Figure 4. There, the red dots represent the starting point, the green dots represent the target point, and the yellow dots represent the meeting node. The step size *m* during the expansion is set to 10. The white grid squares represent the passable area, the black grid squares represent areas with obstacles, and the purple grid squares represent possible meeting nodes.

When the center of the straight-line SG falls within the passable area, the grid where the center is located is the meeting node, as shown in Figure 4a, and the L1 grid is the meeting node. When the center of the straight-line SG falls on an obstacle, a circular area with a step size of *m*_1_ is expanded outward from the coordinates of the midpoint. If there are passable areas within the circular area with a step size of *m*_1_, the substitution values of these passable areas are estimated using the Manhattan distance-calculation formula and the grid with the smallest substitution value is taken as the meeting node, as shown in Figure 4b(i). All rasters from L2 to L9 can be selected as encounter nodes. When there is no passable area within the circular region with a step size of *m*_1_, the step size needs to be increased to expand the circular region with a step size of *m*_2_ outward. If there is a passable area within the circular region with a step size of *m*_2_, the encounter node is selected in the same way. If there is no passable area, the step size is further increased to expand the circular region, as shown in Figure 4b(ii). Grids from L10 to L17 can all be selected as encounter nodes.

#### 2.1.4. Improvements to the Evaluation Function

The BJA* algorithm adds the jump points in the idle area to the Open List through linear SG preprocessing and adds the starting point, target point, and encounter node to the Close List. According to the evaluation function, the node with the lowest cost is selected for expansion. The selection of the evaluation function is crucial to the efficiency of path planning for autonomous vehicles. Therefore, based on different search intervals and different search methods, piecewise evaluation functions are adopted to adapt to the search characteristics of different regions. The traditional A* algorithm integrates heuristic search and cost search and selects the next node to be expanded through an evaluation function. The evaluation function is shown in Equation (1), as follows:

(1) F(n)=G(n)+H(n)

Here, *F*(*n*) is the cost estimation for reaching the target node from the starting node via state *n*; *G*(*n*) represents the actual cost of travel from the starting node to the current node *n*; and *H*(*n*) is a heuristic function used to estimate the cost of travel from the current node *n* to the target node. In path planning on a two-dimensional plane, the Manhattan distance or the Euclidean distance can be used as a heuristic function. The advantage of calculating the Manhattan distance is its high speed, while that of calculating the Euclidean distance is its high accuracy. In a two-dimensional planar coordinate system, let the coordinates of two points be *d_M_* and *d_E_*, respectively. The calculation formulas for Manhattan distance and Euclidean distance are shown in Equations (2) and (3), as follows:

(2) $$d_{M}=\left|x_{1}-x_{2}\right|+\left|y_{1}-y_{2}\right|$$

(3) $$d_{E}=\sqrt{{\left(x_{1}-x_{2}\right)}^{2}+{\left(y_{1}-y_{2}\right)}^{2}}$$

Here, $x_{1}$, $x_{2}$ respectively represent the abscissa of the two points and $y_{1}$, $y_{2}$ respectively represent the ordinate of the two points.

The forward search starts from the starting point S with the goal of reaching the meeting node M. The reverse search starts from the target point G, with the goal of reaching the meeting node M. Suppose the starting point S coordinate is $\left(x_{s},y_{s}\right)$, the ending point G coordinate is $\left(x_{g},y_{g}\right)$, and that during the traversal process of a certain section, the N coordinate of the child node is $\left(x_{n},y_{n}\right)$. The distance *dn* from the child node to the straight-line SG is shown in Equation (4), below:

(4) $$d_{n}=\frac{\left|(x_{g}-x_{s})x_{n}-(y_{g}-y_{s})y_{n}-x_{s}y_{g}+x_{g}y_{s}\right|}{\sqrt{{\left(y_{g}-y_{s}\right)}^{2}+{\left(x_{g}-x_{s}\right)}^{2}}}$$

During the search process in both the forward and reverse directions, a segmented evaluation is conducted with the processed jump points. In the *i* piecewise interval, a penalty term *C_i_*(*n*) is introduced for the heuristic function, which is defined as follows:

(5) $$C_{i}(n)=\beta \cdot e^{\min\left\{\varepsilon ,d_{i}\right\}}$$

Here, β is the penalty factor and ε is a safety threshold indicating that the penalty term will take effect when the distance from the current child node N to the straight-line SG is greater than ε. The larger the value of the penalty term *C*(*n*) when the current child node deviates from the straight-line SG, the more it guides the search acceleration to safely expand in the direction of the straight-line SG. The heuristic function of the *i* segmented interval is as follows:

(6) $$H_{i}(n)=H(n)+C_{i}(n)=H(n)+\beta \cdot e^{\min\left\{\varepsilon ,d_{i}\right\}}\;\;,\;\;\;i=1,2,\text{....},n$$

In summary, the evaluation function of the BJA* algorithm is as follows:

(7) $$F(n)=\left\{\begin{array}{c} G(n)+H_{1}(n)\;\\ G(n)+H_{2}(n)\;\\ \text{......}\;\\ G(n)+H_{n}(n)\; \end{array}\right.$$

### 2.2. DWA Algorithm

#### 2.2.1. Bicycle Model

**Figure 5:**
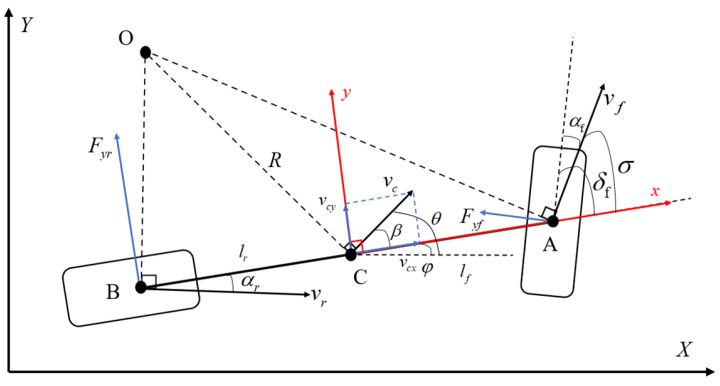
Bicycle model.

The vehicle kinematic model is a model that describes the motion states of a vehicle, such as its position, speed and acceleration. It mainly focuses on the relationships among the vehicle’s position, speed and front-wheel rotation angle without considering the influence of any force. When considering kinematic models, it is generally assumed that the vehicle’s motion is on a two-dimensional plane (*X*, *Y*), the left and right tires of the vehicle have the same steering angle and rotational speed at any time, the motions of the two tires on the left and right can be combined into one tire for description, the vehicle’s traveling speed changes slowly, and the transfer of loads on the front and rear axles is ignored. The vehicle model is simplified into a single-vehicle model [27]. The single model of the vehicle is shown in Figure 5.

Here, *A* represents the center point of the front wheel; *B* represents the center point of the rear wheel; *C* represents the center of gravity of the vehicle; $\delta_{f}$ represents the front-wheel steering angle; $\delta_{r}$ represents the rear-wheel steering angle; $L_{f}$ represents the distance from the center point of the front wheels to the center of gravity of the vehicle; $L_{r}$ represents the distance from the center point of the rear wheel to the center of gravity of the vehicle; *L* represents the vehicle wheelbase, $L=l_{f}+l_{r}$; *V* represents the speed of the vehicle’s center of gravity; β represents the slip angle, the angle between the speed direction of the vehicle’s center of gravity and the longitudinal axis of the vehicle; *O* represents the instantaneous rotation center of the vehicle; *R* represents the trajectory radius of the vehicle; and φ represents the heading angle.

For the further simplification, the vehicle is reduced to a bicycle model with $\delta_{r}=0$, β≅0, and its kinematic equation is as follows:

(8) $$\left\{\begin{array}{l} \overset{\cdot }{X}=V\cos\varphi \\ \overset{\cdot }{Y}=V\sin\varphi \\ \overset{\cdot }{\varphi }=\frac{V\cdot \tan\delta_{f}}{L} \end{array}\right.$$

#### 2.2.2. Speed and Heading-Angle Constraint

The speed constraint of the vehicle is shown in Equation (9):

(9) $$V(v,w)=\tanh(\frac{v_{c}}{v_{\max}})$$

Here, $v_{c}$ represents the average speed of the vehicle’s trajectory and $v_{\max}$ represents the maximum speed of the vehicle.

The hyperbolic tangent function can map the velocity ratio to an interval [−1, 1] and take the absolute value and then restrict it within the interval [0, 1]. When $v_{c}=v_{\max}$, the vehicle is traveling at full speed; when $v_{c}=0$, the vehicle is stationary. The hyperbolic tangent function can prevent sudden score changes at high speeds.

The heading-angle constraints of the vehicle are shown in Equation (10), as follows:

(10) $$A(v,\omega )=1-\frac{\left|\theta_{t}-\theta_{p}\right|}{\theta_{\max}}$$

Here, $\theta_{t}$ is the heading angle of the end of the trajectory; $\theta_{p}$ is the tangent direction of the global path at the start of the trajectory; $\theta_{\max}$ is the maximum heading angle allowed by the path.

Map heading deviations to intervals [0, 1], When the heading deviation is 0, the vehicle is perfectly aligned with the global trajectory, and when the heading deviation is $\theta_{\max}$, the vehicle steers to the limit.

#### 2.2.3. Correction Constraint

The direction-alignment term measures the consistency between the direction of the end point of the vehicle trajectory and the direction of the global target point, and if the end point of the vehicle trajectory points to the target point, the direction alignment is optimal. The constraints for the direction alignment are shown in Equation (11), as follows:

(11) $$A(v,\omega )=1-\frac{\left|\theta_{t}-\theta_{g}\right|}{\pi }$$

Here, $\theta_{t}$ is the heading angle at the end of the trajectory and $\theta_{g}$ is the heading angle of the global target point.

The constraint ensures that the difference in heading angle is within the range [0, π]. Map the angular difference to the interval [0, 1]. When the angle difference is 0, the driving direction is perfectly aligned A=1; When the angle difference isπ, the driving direction is completely opposite A=0.

The safety obstacle-avoidance item quantifies the safe distance between the driving trajectory and the nearest obstacle to ensure that the dangerous area is avoided, and the closer the vehicle is to the obstacle, the higher the safety risk and the lower the score. The constraints of the safety obstacle-avoidance item are shown in Equation (12), as follows:

(12) $$S(v,\omega )=\exp(-\frac{d_{\min}^{2}}{2\sigma_{s}^{2}})$$

Here, $\sigma_{s}$ is the perception-range parameter and $d_{\min}$ is the shortest distance between the vehicle and the obstacle.

The constraint uses the Gaussian function to map the distance to the interval [0, 1]. At $d_{\min}\to \infty$, there were no obstacles around the vehicle; at $d_{\min}\to 0$, the vehicle collided with an obstacle.

#### 2.2.4. Improvement of the Evaluation Function

The evaluation function of traditional DWA usually contains only local information such as speed and steering angle and lacks global path guidance, which makes it easy to fall into local optima. In this paper, the direction-alignment term and distance-detection term are introduced through the vehicle kinematics model, and the global path information is combined with the speed and heading-angle constraint factors to ensure that the final local path is based on the global optimal path. The improved evaluation function is shown in Equation (13), as follows:

(13) $$G(v,\omega )=\sigma \left[\alpha \cdot A(v,\omega )+\mu \cdot S(v,\omega )+\lambda \cdot V(v,\omega )+\eta \cdot \theta (v,\omega )\right]$$

Here, σ is an adaptive smoothing parameter that is dynamically adjusted according to the density of obstacles. α, μ, λ, and η are the weighting coefficients.

The weighting coefficient α is used to control the alignment of the direction of the vehicle, and the value range is [0, 1]. When α=0, the direction alignment is not considered at all; when α=1, the weight of direction alignment is the highest. The weight coefficient μ controls the strictness of obstacle avoidance, and the value range is [0, +∞), with μ=0 representing ignoring obstacle avoidance and increased values of μ indicating increased weight of the safety item. The weighting coefficient λ controls the priority of velocity efficiency, and the value range is [0, 1], with λ=0 indicating ignoring the speed constraint and λ=1 indicating giving priority to the trajectory close to the maximum speed. The weighting coefficient η controls the strictness of the course coordination, and the value range is [0, 1], with η=0 indicating allowing the vehicle to deviate greatly from the path and η=1 indicating strictly maintaining the path direction. All constraint factors are mapped to intervals [0, 1], and the weighting coefficients are dimensionless. This allows constraints of different physical meanings to be directly added to avoid dimensional conflicts.

### 2.3. Fusion Algorithm

**Figure 6:**
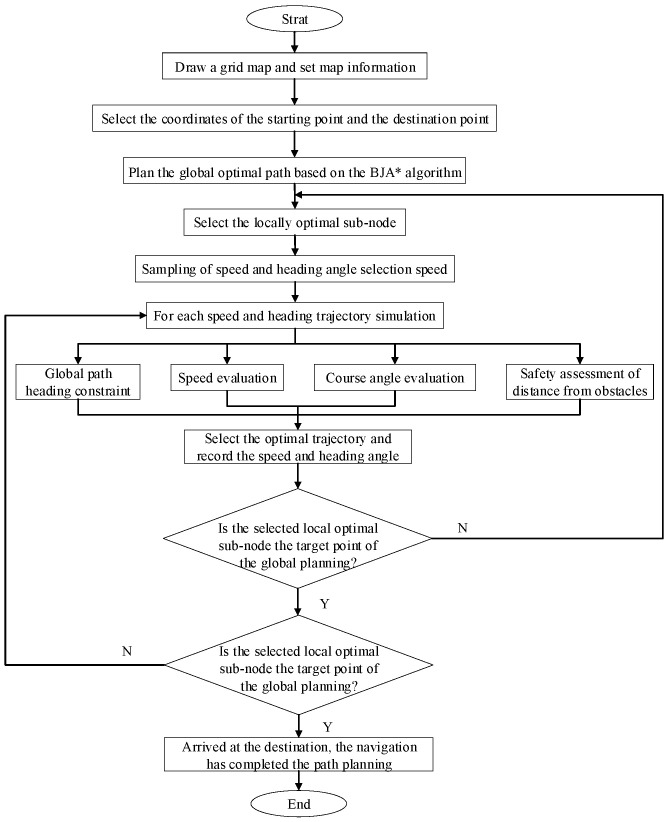
Flow chart of the navigation path-planning algorithm.

This paper integrates BJA* with the improved DWA algorithm, enabling it to achieve global path optimization while also having capabilities for random-obstacle avoidance. The specific steps of the fusion algorithm are as follows. The improved A* algorithm is used to plan the global optimal path. After the global optimal node sequence has been obtained, the improved DWA algorithm is adopted to plan the local path between every two adjacent nodes. The process used by the navigation path-planning algorithm for autonomous vehicles that integrates algorithms is shown in Figure 6.

**Figure 7:**
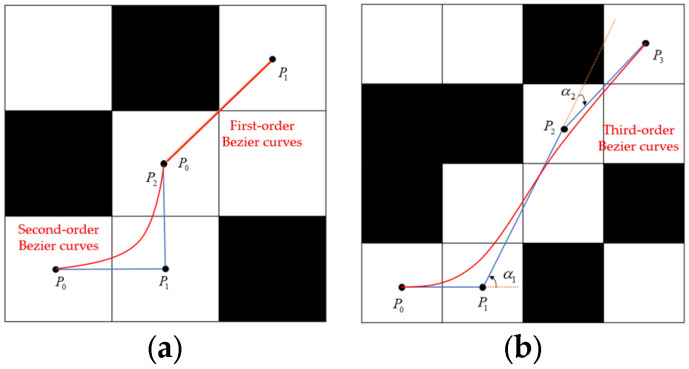
Bezier curves. (**a**) First-order and second-order. (**b**) Third-order.

To ensure consistency between global path updates and local obstacle-avoidance decision-making in the A*–DWA fusion algorithms, the aim is not only to ensure that local obstacle avoidance does not deviate from the global optimal path, but also to allow the global update to adapt to the local dynamic changes in a timely manner, ultimately forming a closed loop of global guidance, local correction, global calibration, and local convergence. This paper uses the following two approaches to address the consistency problem. First, global correction constraints are incorporated in DWA’s local decision-making. Second, the global path is preprocessed to generate a reference path. These approaches mitigates issues such as excessive inflection points and a lack of constraints in the original A* path and provide a clear benchmark for local decision-making. The third-order Bezier curve is used to remove redundant inflection points because the third-order Bezier curve ensures the continuity of the second derivative on the basis of providing shape flexibility and smoothness, making it particularly suitable for paths with large turning angles. The optimized Bezier-curve path is shown in Figure 7.

The Bezier curves can be defined as follows:

(14) $${\left\{\begin{array}{l} B_{1}(t)=(1-t)P_{0}+tP_{1}\\ B_{2}(t)={\left(1-t\right)}^{2}P_{0}+2(1-t)tP_{1}+t^{2}P_{2}\\ B_{3}(t)={\left(1-t\right)}^{3}P_{0}+3{\left(1-t\right)}^{2}tP_{1}+3(1-t)t^{2}P_{2}+t^{3}P \end{array}\right.}_{3}$$

Here, t is the parameter and $P_{1}$, $P_{2}$, and $P_{3}$ are the number of path control nodes.

The above design not only avoids local obstacle-avoidance deviations but also allows the global update to respond to dynamic changes in a timely manner and finally unite global optimization and local obstacle avoidance.

## 3. Results

### 3.1. Global Path-Planning Simulation Verification

#### 3.1.1. Comparison of BJA* Algorithm and Popular A* Improved Algorithm

**Figure 8:**
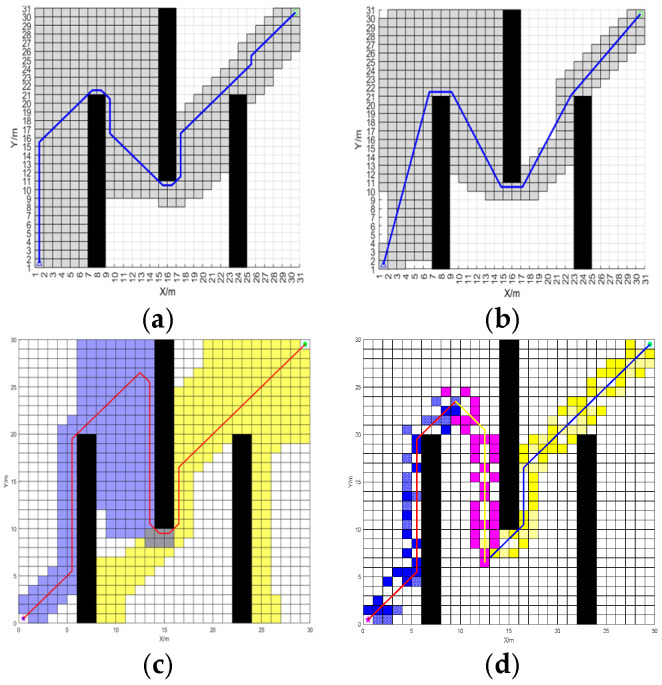
The path-planning results of the A*, Floyd-A*, BA*, and BJA* algorithms on Map 1. (**a**) A*. (**b**) Floyd-A*. (**c**) BA*. (**d**) BJA*.

**Figure 9:**
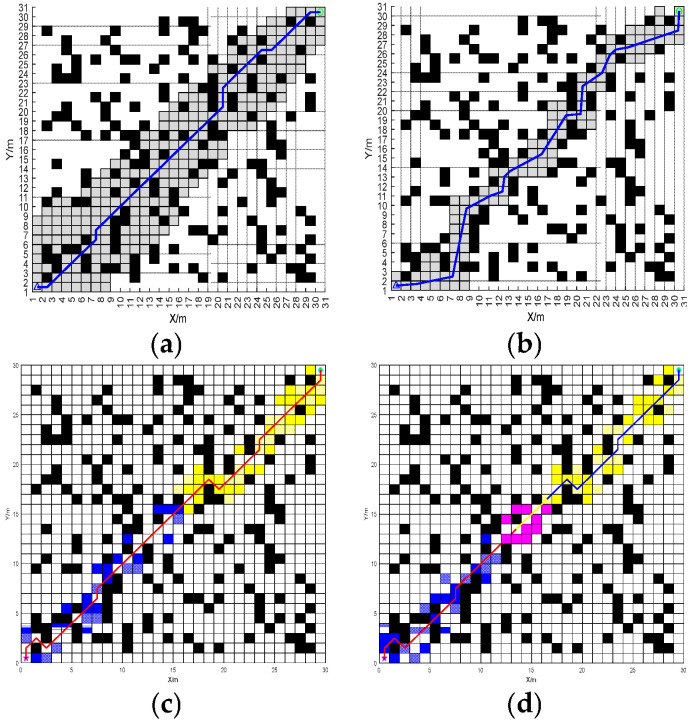
The path-planning results of the A*, Floyd-A*, BA*, and BJA* algorithms on Map 2. (**a**) A*. (**b**) Floyd-A*. (**c**) BA*. (**d**) BJA*.

In order to verify the performance of BJA* algorithm, it is compared in detail with the traditional A* algorithm, the recent popular Floyd-A* algorithm, and the BA* (Bidirectional A*) algorithm under identical maps for path planning. The path-planning results of these four algorithms are shown in Figure 8 and Figure 9.

According to the planning results shown in Figure 7 and Figure 8, the paths planned by algorithms A*, Floyd-A*, BA* and BJA* are analyzed comprehensively based on the three evaluation indicators of path length, algorithm traversal time and path turning times. The simulation data are shown in Table 1.

The path effects of the A*, Floyd-A*, BA* and BJA* algorithms are analyzed. In map 1, compared with traditional A*, Floyd-A* and BA*, the length of the planned path of BJA* is reduced by 17.09%, 12.59%, and 3.29% respectively, and the traversal time of the algorithm is reduced by 93.95%, 23.81%, and 55.97%, respectively. In map 2, compared with traditional A*, Floyd-A* and BA*, the length of the planned path of BJA* is reduced by 7.87%, 5.89%, and 2.22% respectively, and the traversal time of the algorithm is reduced by 89.42%, 88.73%, and 49.78% respectively. In summary, the BJA* algorithm shows significant improvement in path length and algorithm traversal time.

#### 3.1.2. Path Planning Post-Processing

**Figure 10:**
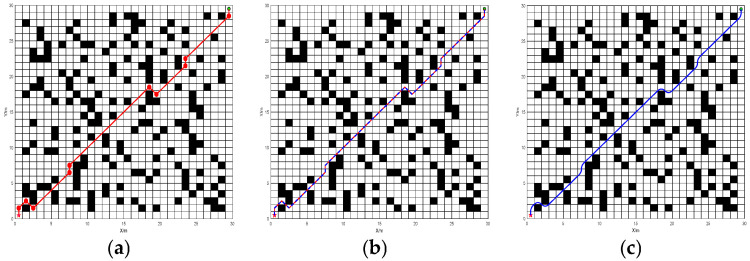
Post-processing of path planning using the BJA* algorithm on Map 2. (**a**) Extraction of key nodes. (**b**) Removal of redundant nodes. (**c**) Path smoothing.

In order to further improve the performance of the BJA* algorithm, the path planned by the BJA* algorithm was processed by extracting key nodes, removing redundant nodes, and smoothing the path. The post-processing result for the BJA* algorithm on Map 2 is shown in Figure 10.

After post processing the BJA*, the path planning result data is shown in Table 2.

According to the data in Table 2, compared with the path without post-processing, the length of the path planned by the BJA* algorithm was reduced by 1.07% and the traversal time of the algorithm was reduced by 12.3% by extracting key nodes and removing redundant nodes. Smoothing the path of BJA* algorithm reduced the length of the path planned by the BJA* algorithm by 6.31%, and the traversal time of the algorithm was reduced by 26.9%. Through the experiment, it can be concluded that the post-processing of the path planned by the BJA* algorithm effectively improves the performance of the BJA* algorithm.

#### 3.1.3. Ablation-Based Analysis of BJA* Algorithm

**Figure 11:**
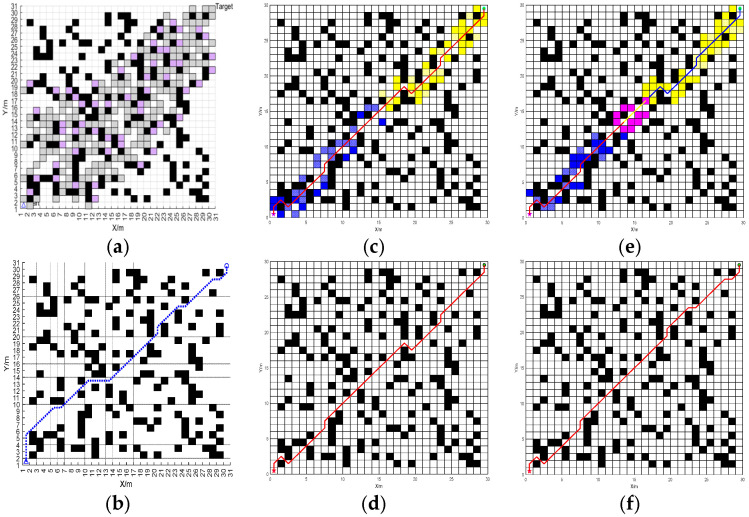
The path-planning results of BJA*-1, BJA*-2, and BJA* on Map 2. (**a**) BJA*-1 ergodic process. (**b**) BJA*-1 planned route. (**c**) BJA*-2 ergodic process. (**d**) BJA*-2 planned route. (**e**) BJA* ergodic process. (**f**) BJA* planned route.

In order to test the effect of each improved part of BJA* algorithm and highlight the contribution of each improved part, an ablation analysis of BJA* was carried out. Since the proposed BJA* algorithm has two improvement components, namely, hop-point optimization search and bidirectional search, it is important to examine the separate effects of these two components and determine which gives the more critical improvement. To examine this effect, we consider two algorithms, BJA*-1 and BJA*-2. BJA*-1 contains only the hop-point optimization search component, while BJA*-2 contains only the bidirectional search component. BJA*-1 and BJA*-2 are tested for the path-planning problem of Map 2. The comparison of the results of path planning with the two algorithms is shown in Figure 11.

The results of the ablation experiments on path planning with BJA* are shown in Table 3.

Through the ablation experiment on the BJA* algorithm, it can be seen that the BJA*-1 algorithm with only the hop-optimization search component reduces the path length by 5.35% and 3.31%, respectively, compared with the A* and Floyd-A* algorithms but has delay amplitudes of 0.87% and 7.37% in the algorithm traversal time. Therefore, it can be judged that the hop-optimization search component plays a role in reducing the length of the planning path in BJA*. Compared with the A* and Floyd-A* algorithms, the BJA*-2 algorithm with only the bidirectional search component reduces the traversal time by 82.8% and 81.7%, respectively, but does not reduce the path length, with 0.16% and 2.31% delay amplitude. Therefore, it can be judged that the bidirectional search component plays a role in reducing the algorithm traversal time in BJA*.

#### 3.1.4. Statistical Test of BJA* Algorithm

**Figure 12:**
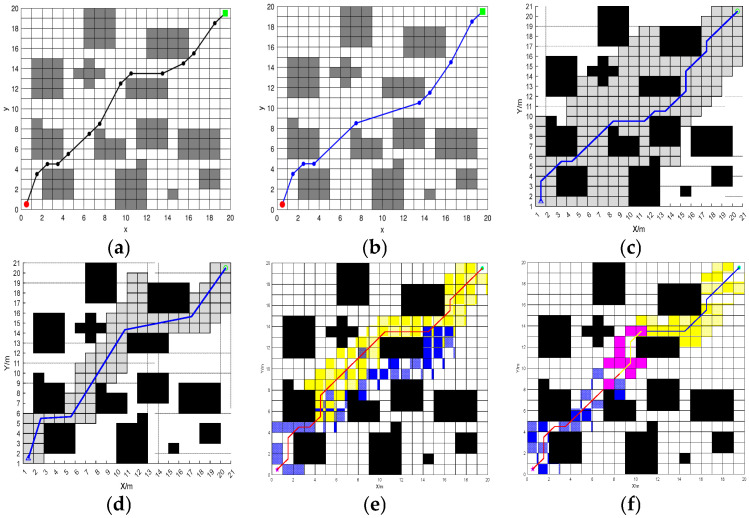
The path-planning results of FWOA, IABC, A*, Floyd-A*, BA*, and BJA* on Map 3. (**a**) FWOA. (**b**) IABC. (**c**) A*. (**d**) Floyd-A*. (**e**) BA*. (**f**) BJA*.

Statistical testing is used to judge whether the data associated with the fusion algorithm in this paper show significant differences. Here, the variance reflects the degree of data discreteness and the confidence interval reflects the uncertainty of the estimate, which can be used to judge whether the improvement is a real effect or a random fluctuation. The IABC algorithm proposed in Ref. [28], the algorithms in [29] include WOA (Whale Optimization Algorithm), PSO (Particle Swarm Optimization), GWO (Grey Wolf Optimizer), STOA (sooty tern optimization algorithm), SSA (Salp Swarm Algorithm), SOA (Seagull Optimization Algorithm), and FWOA (Firefly Whale Optimization Algorithm). In this simulation, 50 iterative experiments are carried out on the algorithm involved in Ref. [29] and the BJA* algorithm proposed in this paper. The Best, Mean, and Optimal Planning Paths are counted multiple times and obtained as shown in Figure 12, and the results of the statistical tests for the path length are shown in Table 4.

For Map 3, the optimal path length planned using BJA* is reduced by 7.68% compared to that planned using A*, by 5.85% compared to Floyd-A*, by 6.54% compared to ABC, by 2.16% compared to IABC, by 2.97% compared to WOA, by 2.16% compared to PSO, by 3.39% compared to GWO, by 5.29% compared to STOA, by 3.39% compared to SSA, by 3.29% compared to SOA, by 2.16% compared to FWOA, and by 6.54% compared to BA*. Differences of 49.70%, 18.35%, 10.19%, 2.15%, 38.63%, 11.89%, 8.66%, 5.23%, 6.50%, 5.25%, 4.97%, and 7.74% were observed in the average length. From the standard deviation, it can be seen that the minimum standard deviation of BJA* proves that its shorter path length is not accidental, but an inevitable result of algorithmic robustness. Other algorithms are either stable but inefficient, or occasionally efficient but extremely unstable, and only BJA* achieves the perfect balance between high efficiency and stability, which is one of its core advantages in unmanned-vehicle path planning.

#### 3.1.5. Comparison of BJA* Algorithm and Fusion Algorithm

**Figure 13:**
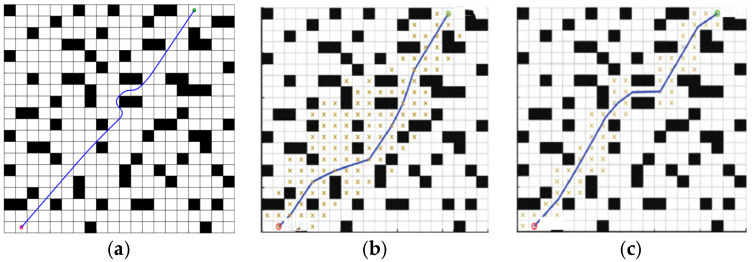
The path-planning results from [30,31], and BJA* on Map 4. (**a**) BJA*; (**b**) [30]; (**c**) [31].

**Figure 14:**
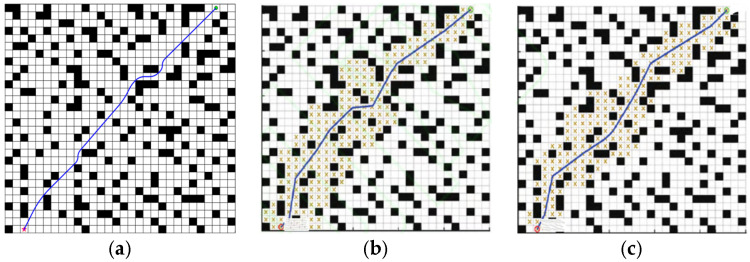
The path-planning results of [30,31], and BJA* on Map 5. (**a**) BJA*. (**b**) [30]. (**c**) [31].

Single optimization algorithms are usually less efficient than mixed algorithms. Therefore, the planning efficiency of the BJA* algorithm proposed in this paper is discussed and compared with that of the fusion algorithm. The fusion algorithms discussed in [30,31] are the latest two improved algorithms for A*, and the fusion algorithms studied in these two papers are compared with the BJA* algorithm mentioned in this paper, respectively, in the same 20 × 20 and 30 × 30 map environments. The planned path is shown in Figure 13 and Figure 14, and the path information is shown in Table 5.

Experimental data analysis shows that the BJA* algorithm performs best in the path-planning task. In Map 4 and Map 5, the path length is shortened by 6.26% and 6.06%, the number of traversal nodes is reduced by 42.59% and 35.26%, and the number of transitions is reduced by 52.63% and 43.75%, respectively, compared with the traditional A*. Compared with the algorithm in literature [30,31], the path length of BJA* is reduced by 3.67% and 6.26%, and the number of node traversals is reduced by 14.80% and 42.59%. Therefore, the BJA* algorithm has significant advantages in reducing redundant calculations and adaptability to a dynamic environment and is suitable for navigation scenarios with high requirements for real-time performance and path quality.

### 3.2. Local Obstacle-Avoidance Simulation Verification

#### 3.2.1. Analysis of Local Obstacle Avoidance by the Fusion Algorithm

**Figure 15:**
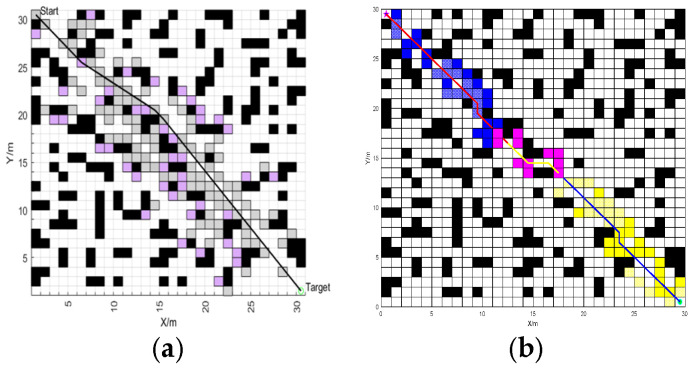
The global path-planning process on Map 4. (**a**) Jump-point selection. (**b**) Bidirectional traversal. (**a**) In “gray squares”, the “gray grids” represent the nodes to be traversed, while the “pink squares” indicate the grids that the jump points will traverse. (**b**) In the figure, “blue squares” represent the traversal from the starting direction, “yellow squares” indicate the traversal starting from the destination direction, and “pink squares” represent the process of determining the meeting nodes.

In order to verify the local obstacle avoidance by the fusion BJA* and DWA algorithm, this paper designs two vehicle-operation scenarios on Map 4: the car-meeting scenario in which the vehicle meets a vehicle traveling in the opposite direction and the scenario in which the vehicle crosses an urban main road with heavy traffic. The length of the path and the linear velocity and angular velocity of the vehicle are used as the evaluation indexes. The global path-planning process for Map 4 is shown in Figure 15.

**Figure 16:**
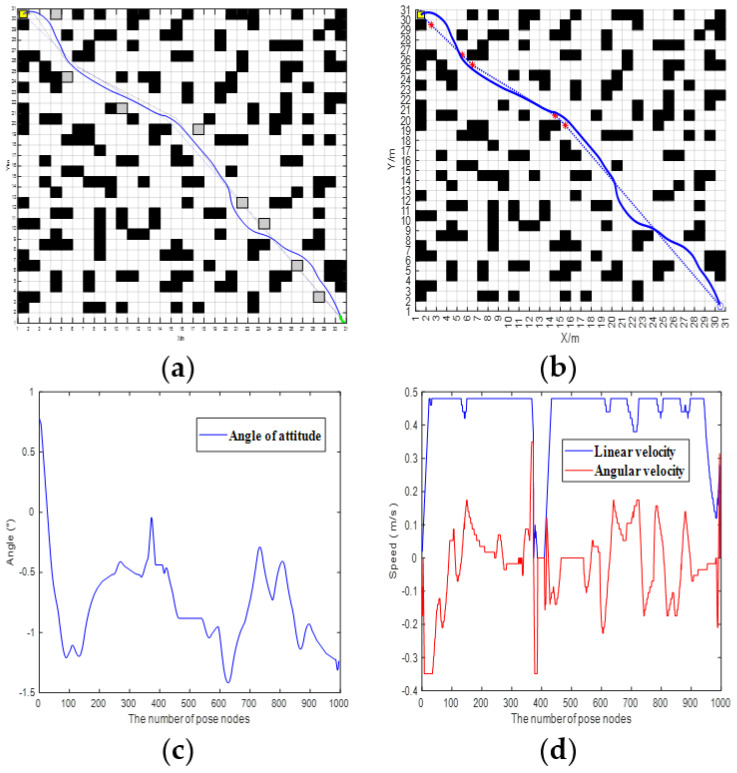
Oncoming vehicles meeting. (**a**) Add dynamic and static obstacles. (**b**) Path trajectory after local obstacle avoidance. (**c**) Changes in the vehicle’s posture angle. (**d**) Linear and angular velocities of the vehicle. In (**a**,**b**), the dotted lines represent the vehicle’s trajectory in the absence of obstacles, while the solid lines represent the actual movement trajectory of the vehicle when avoiding obstacles.

In Scenario 1, the vehicle encounters another vehicle when that vehicle is moving, and the performance of the vehicle in local obstacle avoidance in this scenario is shown in Figure 16.

**Figure 17:**
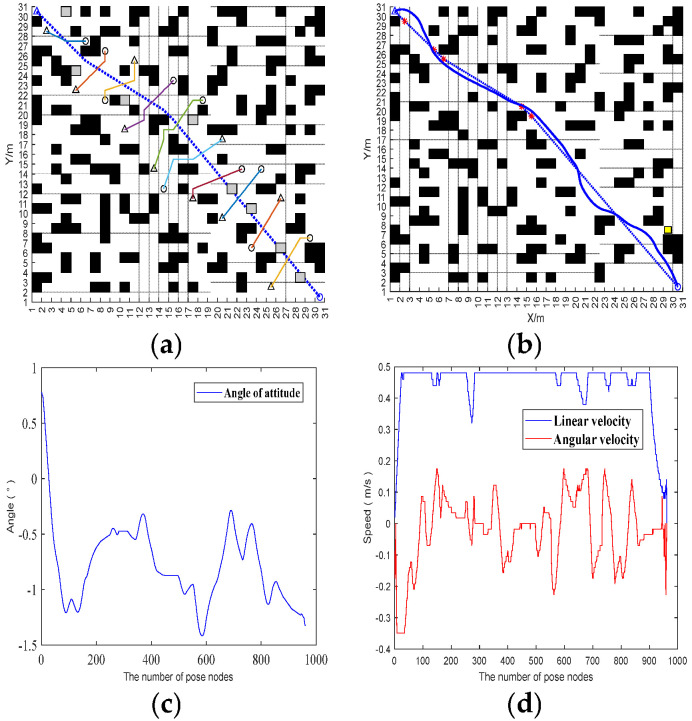
Crossing a congested road. (**a**) Add dynamic and static obstacles. (**b**) Path trajectory after local obstacle avoidance. (**c**) Changes in the vehicle’s posture angle. (**d**) Linear and angular velocities of the vehicle. In (**a**,**b**), the dotted lines represent the vehicle’s trajectory in the absence of obstacles, while the solid lines represent the actual movement trajectory of the vehicle when avoiding obstacles.

In scenario 2, the vehicle crosses a congested road and the performance of the vehicle in local obstacle avoidance in this scenario is shown in Figure 17.

According to the vehicle trajectory in the simulation experiment, when our vehicle and the opposite vehicle are about to meet, our vehicle will stop moving and wait for the opposite vehicle to move, after which our vehicle will continue to move forward. A comparison of the data of the vehicles crossing the road with the vehicles in the opposite direction shows that both of them have large fluctuations in the attitude angle of the vehicles. In Figure 14, the attitude angle quickly drops from 1° to about −1° in the range of 0–100, and there are also many fluctuations in the subsequent period. In terms of linear velocity and angular velocity, Figure 13 shows that linear velocity is mostly maintained at 0.4–0.5 m/s, while angular velocity fluctuates frequently; in the range of 300–500, linear velocity drops briefly and angular velocity changes drastically. Figure 15 shows that when the vehicle is crossing a congested road, the linear velocity also has a brief drop and the angular velocity fluctuates continuously. It can be seen that in different scenes, the vehicle attitude, linear velocity, and angular velocity all change dynamically with the environment and the speed and attitude need to be adjusted frequently when the vehicle is crossing the congested road. It reflects the differing control requirements for vehicle motion under different traffic conditions.

#### 3.2.2. Comparison Test Between Fusion Algorithm and Excellent Algorithm

**Figure 18:**
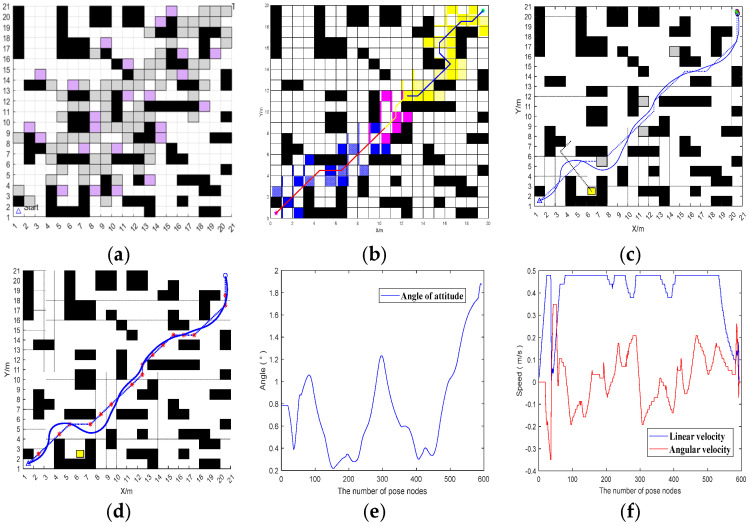
Local obstacle-avoidance performance of the fusion algorithm. (**a**) Jump points to traverse nodes. (**b**) BJA* algorithmic global path planning. (**c**) Set dynamic and static obstacles. (**d**) Local obstacle-avoidance effect. (**e**) Vehicle body angle and attitude. (**f**) Linear velocity and angular velocity. In (**a**,**b**), the dotted lines represent the vehicle’s trajectory in the absence of obstacles, while the solid lines represent the actual movement trajectory of the vehicle when avoiding obstacles.

The local obstacle-avoidance effect of the proposed fusion algorithm is shown in Figure 18.

**Figure 19:**
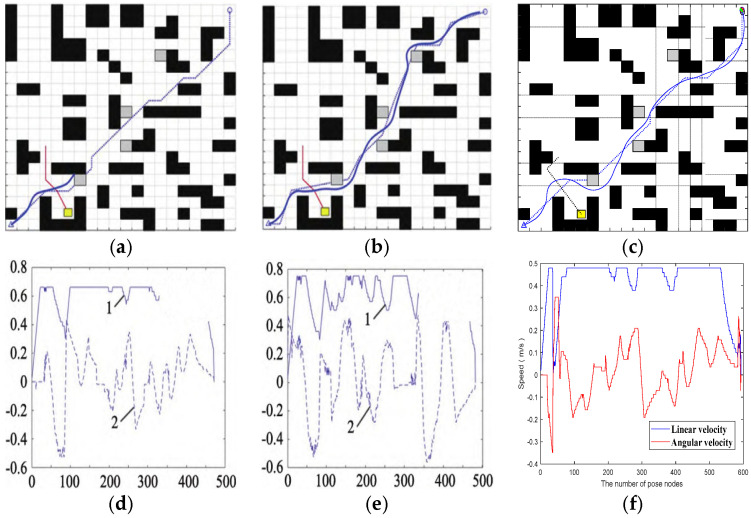
Comparison of the effects of local obstacle-avoidance algorithms. (**a**) An obstacle-avoidance path that integrates A* and DWA. (**b**) The obstacle avoidance path of the fusion algorithm proposed in [32]. (**c**) The obstacle-avoidance path of the fusion algorithm proposed in this paper. (**d**) The speed of the fusion of A* and DWA. (**e**) The speed of the fusion algorithm proposed in [32]. (**f**) The speed of the fusion algorithm proposed in this paper.

On the same raster map, compared with the dynamic and static local obstacle-avoidance performance of the traditional fusion algorithm of A* and DWA and the fusion algorithm of improved A* and DWA proposed in [32], the local obstacle-avoidance effects of the traditional fusion algorithm of A* and DWA and the fusion algorithm of [32] is shown in Figure 19.

Analyzing the speed curves under different algorithms in the figure, it can be seen that the fusion BJA* and DWA algorithm proposed in this paper has obvious advantages in speed. The linear velocity and angular velocity of the traditional A* and DWA fusion algorithms and the fusion algorithm proposed in [32] fluctuate frequently and greatly. For example, the linear velocity of the traditional algorithm fluctuates in multiple segments between 0~0.8 m/s, and the angular velocity also fluctuates greatly. However, the linear speed of the algorithm in this paper is stable at about 0.5 m/s as a whole and only changes briefly at the beginning and end. Although the angular velocity fluctuates, it is relatively smooth. It can be seen that the fusion BJA* and DWA algorithm proposed in this paper can make the device run more smoothly, and the stability of speed control is far superior to those of the other two algorithms.

## 4. Discussion

Aiming to address the global path planning and local dynamic obstacle-avoidance requirements of autonomous vehicles in a structured road environment, this study proposes a hybrid path=planning scheme that integrates and improves the BJA and DWA algorithms. The BJA algorithm significantly expands the search range and optimizes the path smoothness by introducing a 24-neighborhood search, two-way skipping-point strategy, and segmented evaluation function. Experimental results show that the proposed scheme effectively improves the planning efficiency and adaptability in dynamic environment while ensuring that the length of the path is close to the theoretical shortest distance. In the global planning comparison experiment, compared with the traditional A algorithm, the improved BJA algorithm reduces the number of path search nodes by about 85% and optimizes the number of turns by more than 60%, and the planning time is only 15% that of the traditional A algorithm. In addition, in the simulated dynamic meeting and congestion scenarios, the fusion algorithm significantly improves the path smoothness and driving safety compared with the traditional A-plus-DWA method. In this study, the effectiveness of the proposed algorithm is verified by simulation experiments in a two-dimensional raster map environment. There, 2D raster maps are simplified and abstract versions of real-world driving scenarios, where static obstacles simulate impassable objects such as vehicles and barricades, while dynamic obstacles represent moving targets such as pedestrians and vehicles. However, we acknowledge that current MATLAB-based simulations have limitations. A key simplification in this study is the treatment of the vehicle model as a particle, which intentionally abstracts the actual dimensions of the vehicle, kinematic constraints, and precise control responses. This abstraction is a common practice in basic path-planning research used to verify the core logic. In order to fully demonstrate the engineering applicability and reliability of this scheme, further verification on the real vehicle platform or in a simulation environment based on high-fidelity physics, such as CarSim/PreScan, is essential. This forms the core of our future research. Future research will focus on bridging the gap between current simulation and real-world applications. In addition, we recognize that current research on the treatment of dynamic obstacles is still relatively basic. The next step is to extend this framework into more complex, dynamic scenarios. This will involve introducing more accurate environmental awareness and obstacle-trajectory-prediction modules and further optimizing the synergy mechanism between local and global planners to create a complete system capable of safely navigating in highly dynamic, uncertain traffic environments. To this end, we plan to deploy the algorithm on a real vehicle platform equipped with multi-line lidar and vision sensors. The next phase of work will focus on integrating real-time perception data for more accurate scenario understanding and decision planning.

## 5. Conclusions

The core aim of the large-scale implementation of autonomous driving technology is to solve the collaborative problem of global path optimality and local dynamic adaptability. Traditional global path-planning algorithms such as A* and Dijkstra are ill-suited to adaptation to complex and dynamic traffic environments, while local obstacle-avoidance algorithms such as traditional DWA and APF lack global vision and thus fall into local optima. The separation of the two has become a key bottleneck restricting the safe and efficient operation of autonomous driving in real scenarios such as urban congestion and meeting vehicles traveling in the opposite direction. Through the deep optimization and fusion of global planning and local obstacle-avoidance algorithms, a set of path planning schemes considering efficiency, safety, and robustness is formed. Its work value and application potential can be explained from three perspectives: technical innovation, practical application, and future expansion. Through the collaborative optimization and integration of the BJA* global path-planning algorithm and the improved DWA local obstacle-avoidance algorithm, this study effectively solves the core problems of low global efficiency, susceptibility to local optima, and poor dynamic adaptability in traditional path planning for autonomous driving. From the technical perspective, this research enriches the fusion-strategy framework for path planning for autonomous driving and provides a reference technology path for global and local collaborative optimization. From the application perspective, the excellent performance of the algorithm in the complex dynamic environment of the city provides key technical support for L4-level and higher autonomous driving and has significant engineering value in improving driving safety, efficiency, and the user experience. In the future, through parameter adaptation, multi-agent cooperation, extreme-environment resilience and multi-objective optimization, the application scope and performance of the algorithm will be further expanded, providing a more comprehensive solution for autonomous driving and path planning in multi-field mobile platforms.

## Figures and Tables

**Table 1:** Simulation Data.

Algorithm		Path Length (m)	Algorithm Traversal Time (s)	Number of Path Turns (Time)
Map 1	A*	75.598	2.7743	11
	Floyd-A*	71.0122	0.22024	6
	BA*	64.1838	0.38109	9
	BJA*	62.0698	0.16779	5
Map 2	A*	47.7696	1.10756	13
	Floyd-A*	46.7656	1.04056	18
	BA*	45.0122	0.23349	10
	BJA*	44.0122	0.11725	10

**Table 2:** Simulation Data.

Algorithm	Path Length (m)	Algorithm Traversal Time (s)	Number of Pat Turns (Time)
BJA*	44.0122	0.11725	10
Remove redundant nodes	43.542	0.10286	8
Path smoothing processing	41.236	0.08561	5

**Table 3:** Simulation Data.

Algorithm	Path Length (m)	Algorithm Traversal Time (s)	Number of Path Turns (Time)
A*	47.7696	1.10756	13
Floyd-A*	46.7656	1.04056	18
BJA*-1	45.2162	1.11725	12
BJA*-2	47.8469	0.19046	11
BJA*	44.0122	0.11725	10

**Table 4:** Simulation Data.

Algorithm	Best	Mean	Std.
A*	30.1658	55.4892	78.965
Floyd-A*	29.5797	34.1859	6.3254
[28] ABC	29.7990	31.0777	0.6451
[28] IABC	28.464	28.5245	0.1818
[29] WOA	28.7003	45.4813	67.0978
[29] PSO	28.464	31.6773	4.6007
[29] GWO	28.8269	30.5589	2.4051
[29] STOA	29.4046	29.4531	0.1345
[29] SSA	28.8269	29.8522	0.3687
[29] SOA	28.8269	29.458	0.2247
[29] FWOA	28.464	29.372	0.5622
BA*	29.799	30.2543	0.5971
BJA*	27.8496	27.912	0.1527

**Table 5:** Simulation data.

Algorithm		Path Length (m)	Traversing the Number of Nodes (Time)	Number of Path Turns (Time)
Map 4	A*	25.79	141	11
	literature [30]	25.25	116	7
	literature [31]	25.04	66	7
	BJA*	24.2787	57	4
Map 5	A*	39.94	230	8
	literature [30]	40.34	213	9
	literature [31]	38.92	184	8
	BJA*	37.336	156	5

## Data Availability

The original contributions presented in this study are included in the article. Further inquiries can be directed to the corresponding author(s). Data is not made public.
